# The increase in intensity and frequency of surface air temperature extremes throughout the western South Atlantic coast

**DOI:** 10.1038/s41598-023-32722-1

**Published:** 2023-04-25

**Authors:** Fábio H. C. Sanches, Fernando R. Martins, William R. P. Conti, Ronaldo A. Christofoletti

**Affiliations:** Institute of Marine Science, Federal University of São Paulo (IMar/UNIFESP), Santos, SP 11070-102 Brazil

**Keywords:** Climate sciences, Atmospheric science, Climate change, Environmental sciences, Environmental impact

## Abstract

The climate is changing. At this stage, it is important to specify an ‘extreme’ climate and identify patterns that indicate its potential harm worldwide, including the coastal zones. Herein, we considered extremes based on the “Peaks Over Threshold” method from the “Extreme Value Theory”. We looked after geographical patterns of surface air temperature (SAT) extremes (e.g., T_max_, T_min_, daily temperature range (DTR), and inter-daily temperature range) over the last 40 years throughout the Brazilian coast. Overall, we found a trend increase in intensity and frequency, but the duration was barely affected. The latitudinal pattern of extremes and the temperatures considered extremes followed the settled perception that areas in higher latitudes will be more affected by the extent of warming. Additionally, the seasonal pattern of DTR demonstrated to be a good approach to make inferences about air mass changes, but joint analyses on extremes with other atmospheric variables are desirable. Given the potential effects of extreme climates on society and natural systems over the world, our study highlights the urge for action to mitigate the effects of the increase in SAT in coastal zones.

## Introduction

Climate change is a reality and a major threat to humankind. The Earth´s global mean temperature is increasing over the past few decades and will continue in the following years^1^. Although the mean climate is a useful parameter to investigate climate changes^2^, it is also important to understand the patterns related to climate extremes. An increase in global mean temperature might lead to changes in the intensity, frequency, and duration of these extremes, with pessimistic projections for the future^3–5^. The increase in extreme heat or cold is responsible to affect human health, impacting social and also natural systems over the world^3^. Recently, there has been much research describing the impacts of extreme events^3,6^. At this stage, it is important to specify what an ‘extreme’ is and identify patterns of extremes to understand their potential harms in different regions of the planet.

A critical discussion on climate extremes is the divergence in the concept of ‘extreme’ across a wide range of disciplines^7,8^. It is pivotal that studies are clear on its definition. It can be described by including physical and socioeconomic impacts^7,8^, such as earthquakes^9^, coastal storms and floods^10^, droughts concurrent to heat waves^11^, or the number of frost days^12^. Alternatively, an extreme can be defined based on probabilities of occurrences exceeding a certain threshold^7,8^. In this study, we will consider this last one, determining “extremes” by choosing the threshold based on the “Peaks Over Threshold” (POT) method, from the “Extreme Value Theory”. By this approach, “extremes” are rare events identified over a chosen threshold in the upper tail distribution of a given parameter, a common method applied across different disciplines, such as financial, insurance, hydrology, and environmental sciences^13–16^. An advantage of this approach is that it is possible to make some indexes regarding the intensity, frequency, and duration of extremes. A point to be highlighted is that, in this work, the method in question is applied to the series of deviations from the normalized 40-year monthly mean, not to the original historical series, which allows us to observe differences in extreme values in different months, seasons, and years.

Several studies on climate extremes are focused on surface air temperature (SAT) due to its impacts on society and ecosystems as a whole^12,17,18^. Indeed, it has been observed over the last decade significant intensifications in extreme daily maximum temperatures (T_max_) and daily minimum temperature (T_min_), with the last one associated with marked increases^17,19^. This also might affect the diurnal temperature range (DTR), the thermal amplitude within a daily cycle (i.e., same day T_max_ − T_min_), and the inter-daily temperature range (IDTR), the differences of T_max_ and T_min_ between 2 consecutive days (i.e., T_max_^day^ − T_max_^day+1^; T_min_^day^ − T_min_^day+1^). The use of DTR or IDTR can potentially be used to make inferences about air mass changes, such as the passage of weather fronts since it is expected great temperature variability in a window of 24 h in these cases^20^. Understanding the geographical pattern of SAT (e.g., T_max_, T_min_, DTR, and IDTR) is fundamental since it drives the weather dynamics^21,22^, climate variability^23^, and also affects human health^18^.

Climate change impacts are especially important to coastal zones^24^. The ocean can absorb and store the heat excess, regulating the climate, and reducing the rate of SAT warming^25^. Thus, most studies have been focusing on sea-level rise, coastal erosion, or ocean-related variables^26,27^, and less is understood about the patterns of SAT extremes in coastal areas. The Brazilian coast is a region at potential risk for climate extremes due to its large latitudinal extension adjacent to the Atlantic Ocean, including 5 Marine Ecoregions of the World (MEOW)^28,29^, from tropical to temperate weather^30^. The interacting air masses (cold or warm) can be geographically divided into equatorial, tropical, or polar types, with the Brazilian northern coast influenced by the equatorial North Atlantic and the equatorial South Atlantic air masses, while the tropical and southern coasts are affected by the tropical Atlantic and the polar Atlantic air masses, with different effects depending on the season^30–32^. Given the context, herein we investigated SAT patterns in all 5 MEOW on the Brazilian coast, aiming to (1) identify potential changes in the intensity, frequency, and duration of SAT climate extremes over the last 40 years (e.g.: T_max_; T_min_, DTR and ITDR); (2) evaluate the seasonal patterns of extremes; (3) specify monthly temperatures considered extremes for each location; and (4) evaluate DTR and IDTR as indicators of air masses exchange, such as during passage of weather fronts.

## Methods

### Climate data

We have used historical surface air temperature (SAT), from both observed and reanalysis datasets, from five locations throughout the Brazilian coast, with at least one weather station on each Marine Ecoregion of the World (MEOW). The locations were chosen based on the availability of good quality observed dataset and the proximity to the closest grid point from the reanalysis data. From north to south, the chosen locations were: São Luís/MA; Natal/RN; São Mateus/ES; Iguape/SP; and Rio Grande/ RS.

The observed meteorological dataset was obtained from automatic weather stations managed by the Brazilian National Institute of Meteorology (INMET; https://portal.inmet.gov.br/, latest access 01 October 2020). Hourly air temperature data were obtained from 01/01/2007 to 12/31/2018 in each location. See Ref.^33^ for the observed dataset used herein. The reanalysis was the ERA5 reanalysis dataset from the European Centre for Medium-Range Weather Forecasts (ECMWF) within the Copernicus Climate Change Service (C3S) (ref: https://doi.org/10.24381/cds.adbb2d47). The atmospheric reanalysis systems aim to assimilate irregular observational data acquired over an extended period in the past to feed a numerical weather forecast model to reconstruct the weather and meteorological phenomena through a uniform grid with spatial homogeneity, temporal continuity, and a multidimensional hierarchy, becoming a valuable data source to study weather systems and climate variability. The ERA5 is the fifth generation ECMWF atmospheric reanalysis and it combines vast amounts of historical observations into global estimates using advanced modeling and data assimilation systems to provide hourly estimates of a large number of atmospheric, land, and oceanic climate variables^34^. Recent works used the ERA5 reanalysis system to evaluate atmospheric data including downward solar radiation, precipitation, and temperature^35–38^. Data obtained were hourly SAT (2 m) from 01/01/1980 to 12/31/2019 for the closest grid point to the observed weather station (grid scale of 0.25° × 0.25°). See Ref.^39^ for the reanalysis dataset used herein.

A reliable long-term time series is required to investigate extreme climate events. The ten-year dataset acquired at automated weather sites (AWS) must be at least 30 years long to provide reliable representativeness of the contribution of low-frequency weather phenomena to extreme climate extreme patterns in the five coastal locations. Generally, the temperature data series provided by the reanalysis databases are long enough but they present bias due to misrepresenting the local weather patterns in the numerical models, as ERA-5^40,41^. Combining model outputs and ground measurements is a standard methodology to overcome the time-lengthening limitation^42^. For this research, we found the linear regression bias removal method suitable to get a reliable and representative long-term dataset for each coastal location. The bias removal using a linear regression model aims at finding a linear relationship between the measured and modeled data, which often can result in an improved coefficient of determination of the pair of random variables^42^. Therefore, herein the bias correction was made in all reanalysis time series, based on the comparison of the 12 years of observed data to the reanalysis data from the same period. Important to mention that the altitude data of the ERA-5 grid points were similar to the altitude of AWS locations in all five sites, so we assumed the bias between modeled and measured data are related primarily to uncertainties in numerical parameterizations in ERA-5. See Ref.^43^ for all observational and reanalysis data comparison and bias adjustments results; and Ref.^44^ for the scripts codes.

### Statistical analyses of climate extremes

The extreme analysis requires a stationary time series. The first step was to remove the trend and seasonality from the site-adapted SAT time series $X(i)$, where *i* = (*Year* (*Y*), *Month* (*M*), *Day* (*D*), *hour* (*h*)).

The SAT time series are represented by additive models, meaning that the original data is the sum of a trend, a seasonal component, and a residual part: ($X(i) = trend(i) + seasonal\,component(i) + residual(i)$). The detrended time series (*Z*) is obtained by subtracting from $X(i)$ its 12 months moving average ($Z(i) = X(i){-}\overline{X(i)}_{ma}$). Next, to construct a deseasonalized time series ($W(i)$), we subtract the monthly average $\overline{Z( \cdot ,M, \cdot , \cdot )}$ from each value $Z( \cdot ,M, \cdot , \cdot )$ and divide by the standard deviation $\sigma ( \cdot ,M, \cdot , \cdot )$, as shown in Eq. (1). The twelve-monthly values for $\overline{Z( \cdot ,M, \cdot , \cdot )}$ and $\sigma ( \cdot ,M, \cdot , \cdot )$ are calculated taking into account the entire period from 1980 until 2019^45^:

1 $$W(Y,M,D,h) = \frac{{Z(Y,M,D,h) - \overline{Z( \cdot ,M, \cdot , \cdot )} }}{\sigma ( \cdot ,M, \cdot , \cdot )}$$

We also evaluated the stationarity of the time series using the augmented Dickey–Fuller (ADF) test and checked the autocorrelation function for the time series to assess the degree of dependence in the data by performing a Ljung–Box Test^46^.

To identify SAT extreme values, we used the “Extreme Value Theory”. It consists in extracting from a continuous record the peaks of values that exceed a certain threshold, referred to as the "Peak over threshold" method (POT). Herein we applied the POT method based on the Generalized Pareto Distribution (GPD), to describe the upper tail behavior from data distribution, providing good extrapolation for exceedances over a threshold^15,16^. Once a threshold is determined, all greater values are considered for the analysis. For a fixed threshold *u*, we construct the series of exceedances *W*_*i*_* − u,* as indicated in Eq. (2).

2 $$E(u) = \{ Wi - u,i \in D:Wi > u\} = \{ Wi - u,i \in \varepsilon (u)\} ,$$

wherein *ε*(*u*) denotes the subset of indexes in *D* for which *W*_*i*_ > *u*. For *u* large enough, the distribution of exceedances above *u* is approximated by a GPD G_ξ,σu_(*x − u*) describe in Eq. (3).

3 $$G_{\xi ,\sigma u} \left( {x - u} \right) = Pr\left( {X < x|X > u} \right) = \left\{ {\frac{{1 - \left[ {1 + \xi \left( {\frac{x - u}{{\sigma u}}} \right)} \right]^{{\frac{ - 1}{\xi }}} \,{\text{if}}\,\upxi \ne {0}}}{{1 - exp\left[ { - \left( {\frac{x - u}{{\sigma u}}} \right)} \right]\,{\text{if}}\,\upxi = {0}}}} \right.,$$

wherein *ξ* and *σ*_*u*_ are shape and scale parameters, respectively, $x \ge uif\xi \ge 0 \wedge u \le x \le u - \frac{{\sigma_{u} }}{\xi }if\xi < 0$.

We used two graphical methods to select an appropriate threshold, neither too high to get enough observations, nor too low to avoid non-extreme values. The first one is the mean residual life plots: for a range of thresholds *u*, we identified the corresponding mean threshold excess, plotted this mean threshold excess against *u*, and looked for the value *u*_0_ above which we can see linearity. If the GPD assumption is correct, then the plot should be linear before it becomes unstable due to the few very high data points. The second graphical method was the parameter stability plot: if the exceedances of a high threshold *u*_0_ follow a GPD with parameters *ξ* and *σ*_*u*_, for any threshold *u* such that *u* > *u*_0_, the exceedances still follow a GPD with shape parameter *ξ*_*u*_ = *ξ* and scale parameter *σ*_*u*_ = *σ*_*u0*_ + *ξ* (*u − u*_0_)*.* Thus, the thresholds were chosen at the value where the shape and scale parameters remained constant^15,16^.

### Response variables and extreme indicators

From the extreme analysis for each location, we decluttered our SAT hourly dataset (independent threshold excesses with autocorrelation checked by Ljung–Box Tests) into response variables that would be used to build statistical indicators:

1. T_max_: Daily maximum temperatures;
2. T_min_: Daily minimum temperatures;
3. DTR: Daily temperature range (i.e., T_max_ − T_min_ from the same day);
4. + IDT_max_R: Positive inter-daily T_max_ range (i.e., differences of T_max_ on consecutive days: the following day has lower T_max_ than the previous one);
5. + IDT_min_R: Positive inter-daily T_min_ range (i.e., differences of T_min_ on consecutive days: the following day has lower T_min_ than the previous one);
− IDT_max_R: Negative inter-daily T_max_ range (i.e., differences of T_max_ on consecutive days: the following day has higher T_max_ than the previous one);
− IDT_min_R: Negative inter-daily T_min_ range (i.e., differences of T_min_ on consecutive days: the following day has higher T_min_ than the previous one).

We considered an “extreme occurrence” as any situation that exceeds a certain threshold, regardless of what happens the day before or after. On the other hand, an “extreme event” is related to the number of consecutive days with an extreme occurrence (in other words, related to its duration, i.e., 1-day event, 2-day event, …, n-day event). Thus, from each response variable, we used the following extreme indicators to access the intensity, frequency, and duration of extreme occurrences and extreme events:

1. Exceedances over the threshold series (i.e., the intensity of extreme occurrences);
2. Time elapsed between consecutive extreme occurrences (i.e., the frequency of extreme occurrences);
3. Number of extreme occurrences per year (complements the frequency of extreme occurrences);
4. Time elapsed between consecutive extreme events (i.e., the frequency of extreme events);
5. Number of extreme events per year (complements the frequency of extreme events);
6. Number of days per extreme event (i.e., the duration of extreme events).

### Trend analyses

We performed a series of Mann–Kendall trend tests to identify whether the intensity, frequency, and duration of the extreme occurrences/ events are increasing over time. This is a robust test widely used for time series data analysis in areas of atmospheric and oceanic studies^47^. We calculated all extreme indicators from each response variable to all locations, whether there are consistently increasing or decreasing trends through time in values exceeding the established threshold. Values were considered statistically significant when *p* < 0.05.

### Seasonal frequency of extremes and monthly temperatures overview

Finally, we also tested for each response variable from all locations the monthly frequency of extreme occurrences and events in the whole period to understand seasonal patterns. In addition, the month-wise temperature boxplots are presented to have insights into extreme temperatures. Thus, with a joint analysis of all of our results, it allows us to: understand if the extreme temperatures have been more intense, frequent, and long-lasting throughout the last 40 years along all 5 Marine Ecoregions on the Brazilian coast; understand the seasonal pattern of extremes; specify temperatures considered extremes per month for each location; and evaluate if DTR and inter-daily SAT variability can be good indicators to make inferences about changes in air masses, such as during passage of weather fronts.

All data analyses were performed using Python language^48^, version 3.7.11. In all Figures, graphs were plotted with Matplotlib^49^, maps with ‘ggplot2’ package^50^ from R software^51^, and edited in Inkscape software (https://inkscape.org/).

## Results

### Trends in intensity, frequency, and duration of extremes

**Figure 1:**
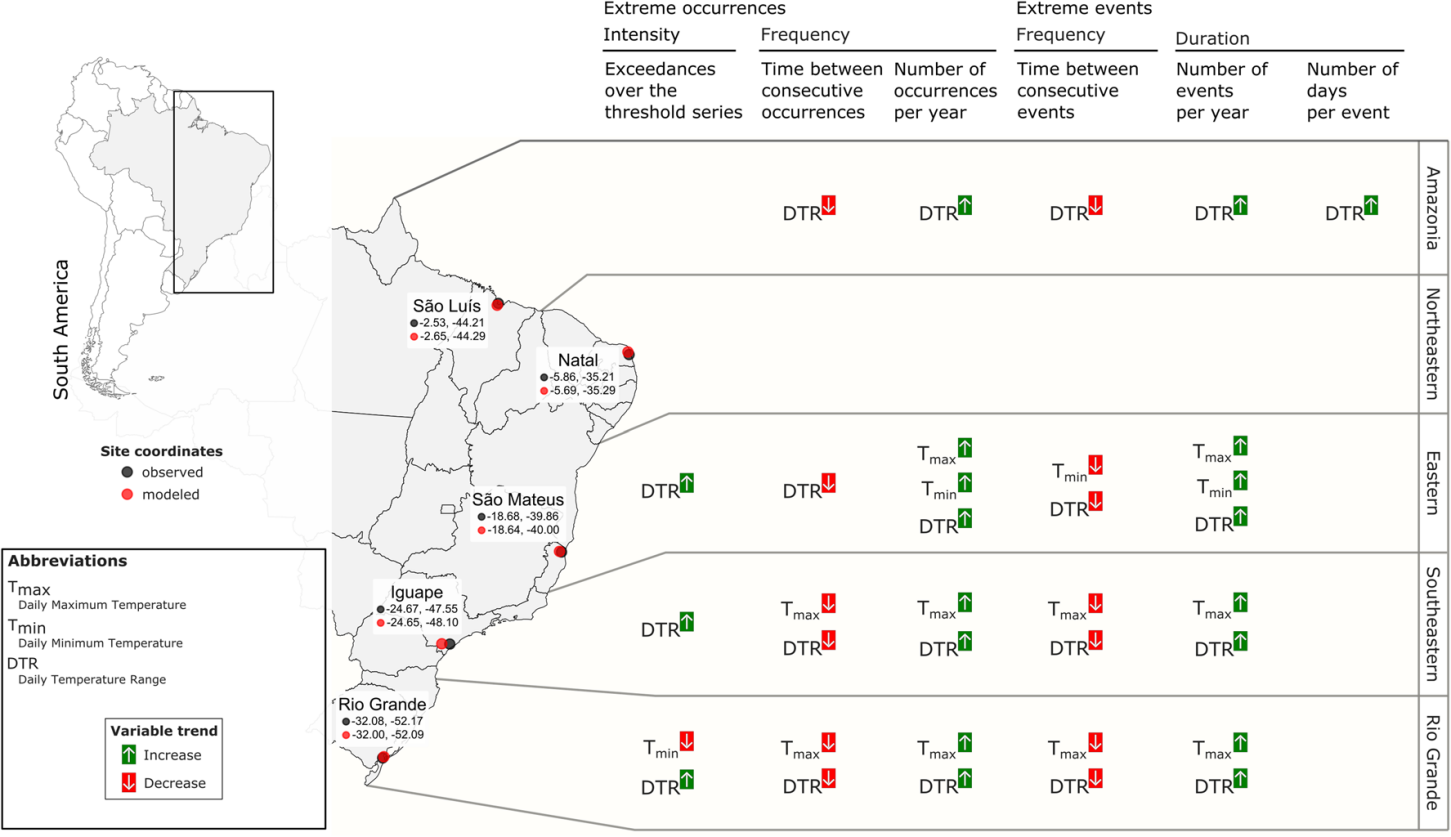
Overall statically significant results from trend analysis to all 5 MEOW in the last 40 years. The results are presented to the following response variables: Daily maximum temperatures (T_max_); Daily minimum temperatures (T_min_); and Daily temperature range (DTR), with their respective extreme indexes that indicate intensity (Exceedances over the threshold series), frequency (both Time elapsed between consecutive extreme occurrences and events, as well as the Number of extremes per year), and duration (Number of days per extreme event).

**Figure 2:**
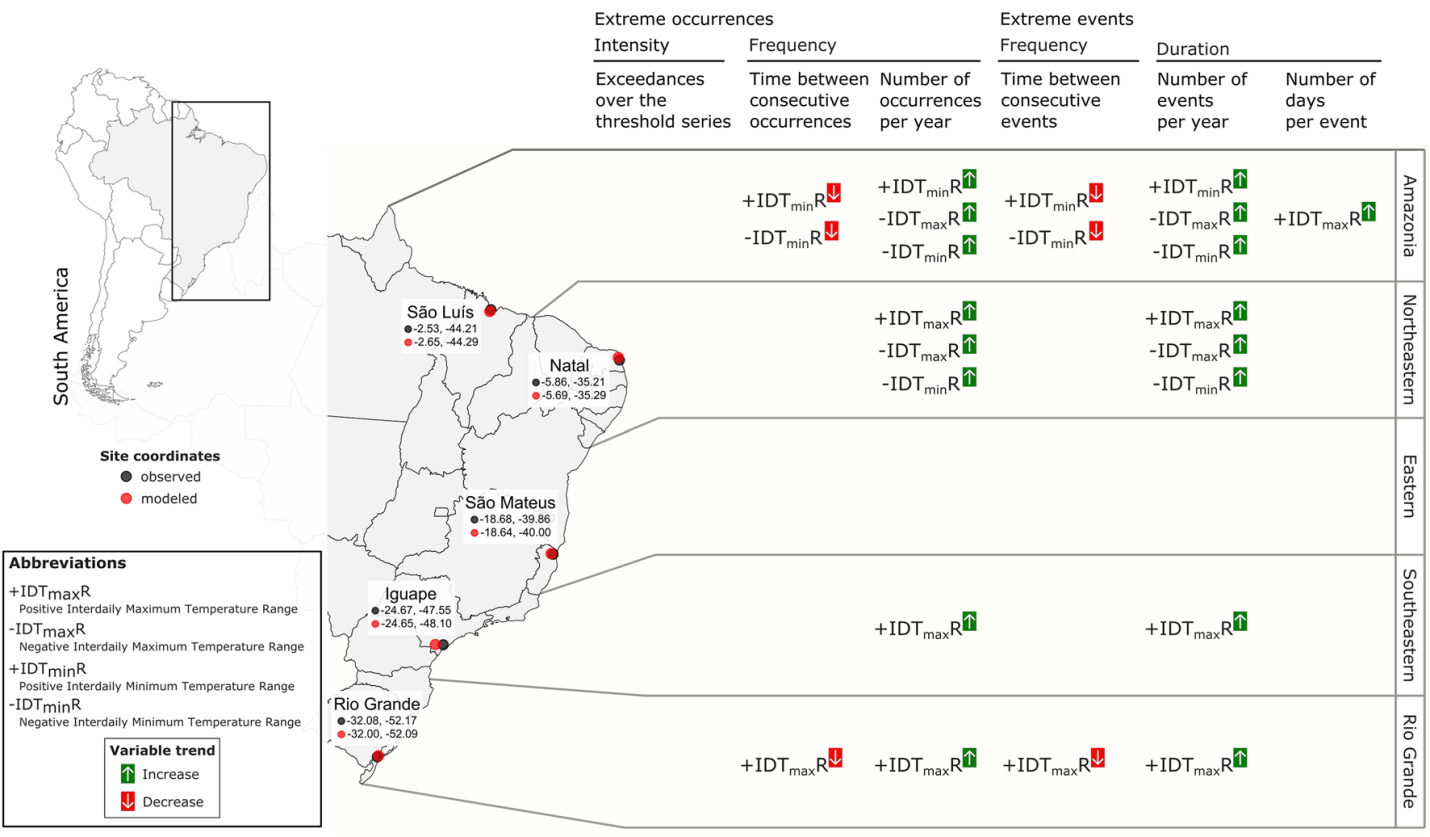
Overall statically significant results from trend analysis to all 5 MEOW in the last 40 years. The results are presented to the following response variables: positive inter-daily T_max_ range (+ IDT_max_R); positive inter-daily T_min_ range (+ IDT_min_R); negative inter-daily T_max_ range (− IDT_max_R); and Negative inter-daily T_min_ range (− IDT_min_R), with their respective extreme indexes that indicate intensity (Exceedances over the threshold series), frequency (both time elapsed between consecutive extreme occurrences and events, as well as the number of extremes per year), and duration (Number of days per extreme event).

The results are presented herein as a comparison between all 5 Marine Ecoregions for each response variable (see Fig. 1: T_max_, T_min_, and DTR; Fig. 2: + IDT_max_R, + IDT_min_R, − IDT_max_R, and − IDT_min_R), as well as extreme indexes that indicate intensity (Exceedances over the threshold series), frequency (both Time elapsed between consecutive extreme occurrences and events, and also the Number of extremes per year), and duration (Number of days per extreme event). See Ref.^52^ for the detailed results per region that can be of interest to local researchers or decisions makers; and Ref.^53^ for the scripts codes.

From the northern to the southern coast, São Luís climate extremes occurrences and events in T_max_ and T_min_ have not increased in intensity, frequency, and duration throughout time, similarly to what was observed in Natal. Alternatively, São Mateus demonstrated an increasing trend in the frequency of extreme occurrences and events of both T_max_ and T_min_; Iguape showed an increasing trend in the frequency of extreme occurrences and events to T_max_; and Rio Grande showed not only an increasing trend on the frequency to both occurrences and events of T_max_ but also a decreasing trend in the intensity of T_min_ (i.e., extremes of T_min_ are getting warmer through time).

We found a different pattern concerning the climate amplitude on the same day (DTR) or in between consecutive days (IDTR: + IDT_max_R, + IDT_min_R, − IDT_max_R, and − IDT_min_R). In São Luís, extremes are becoming more frequent to the DTR, and to some IDTRs that indicate both cooling or warming situations (+ IDT_min_R and − IDT_min_R, respectively). In addition, this was the only location that presented an increase in the duration of extreme events, in both DTR and + IDT_max_R (i.e., cooling situation). In Natal, we only observed an increase in the frequency of extreme events and occurrences to some IDTR that indicates both cooling or warming situations (+ IDT_max_R and − IDT_min_R, respectively). Going southward, São Mateus, Iguape, and Rio Grande had not only an increase in the frequency of the DTR but also in its intensity. In contrast, there was no effect on any extreme indicators related to the inter-daily SAT variation in São Mateus, while Iguape and Rio Grande showed an increasing trend in the frequency of extreme occurrences and events only for one cooling situation (+ IDT_max_R).

### Seasonal overview on extremes frequency

**Figure 3:**
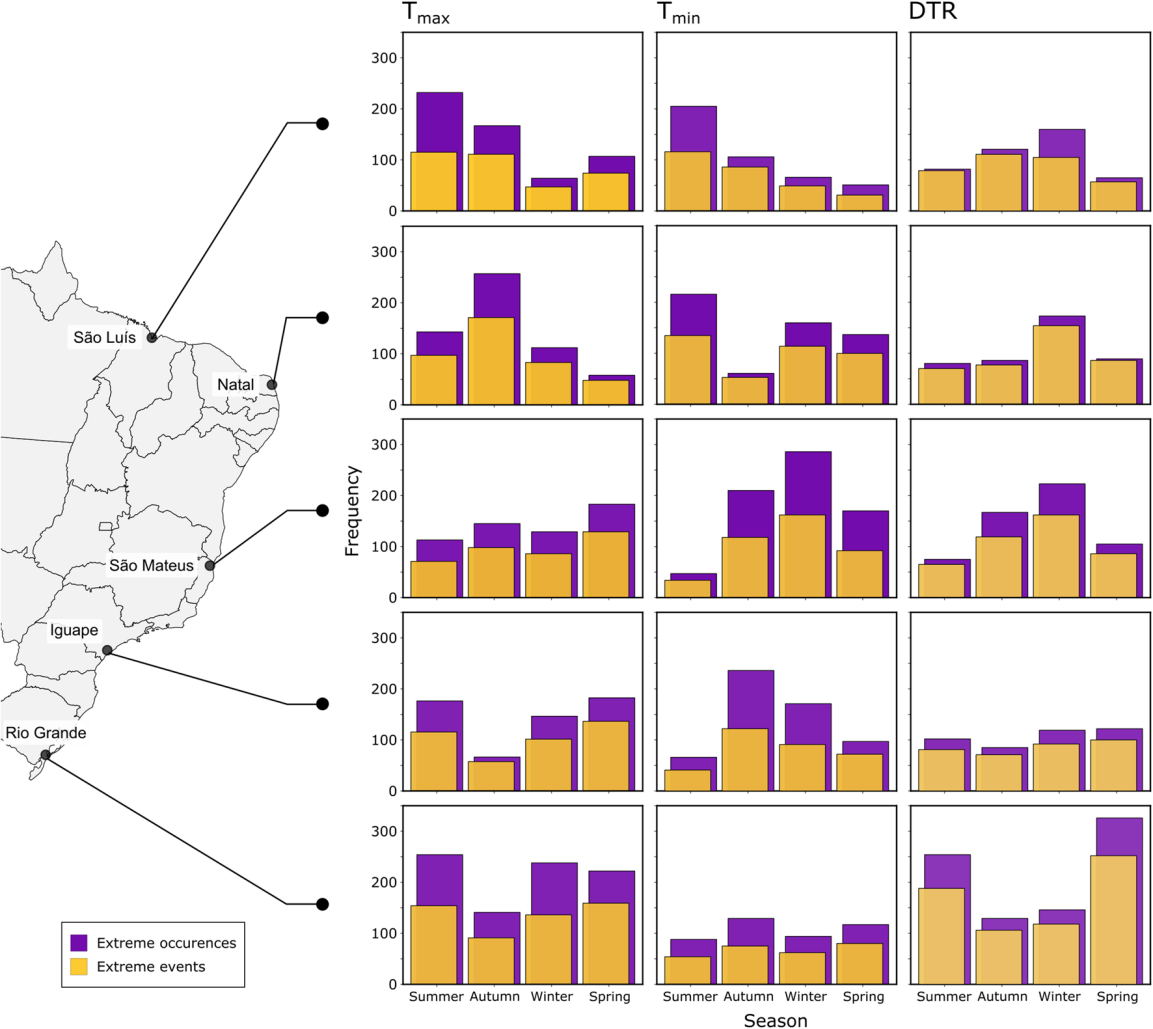
Overall seasonal frequency of extreme occurrence and events from all 5 MEOW in the last 40 years. The results are presented to the following response variables: daily maximum temperatures (T_max_); daily minimum temperatures (T_min_); and daily temperature range (DTR).

**Figure 4:**
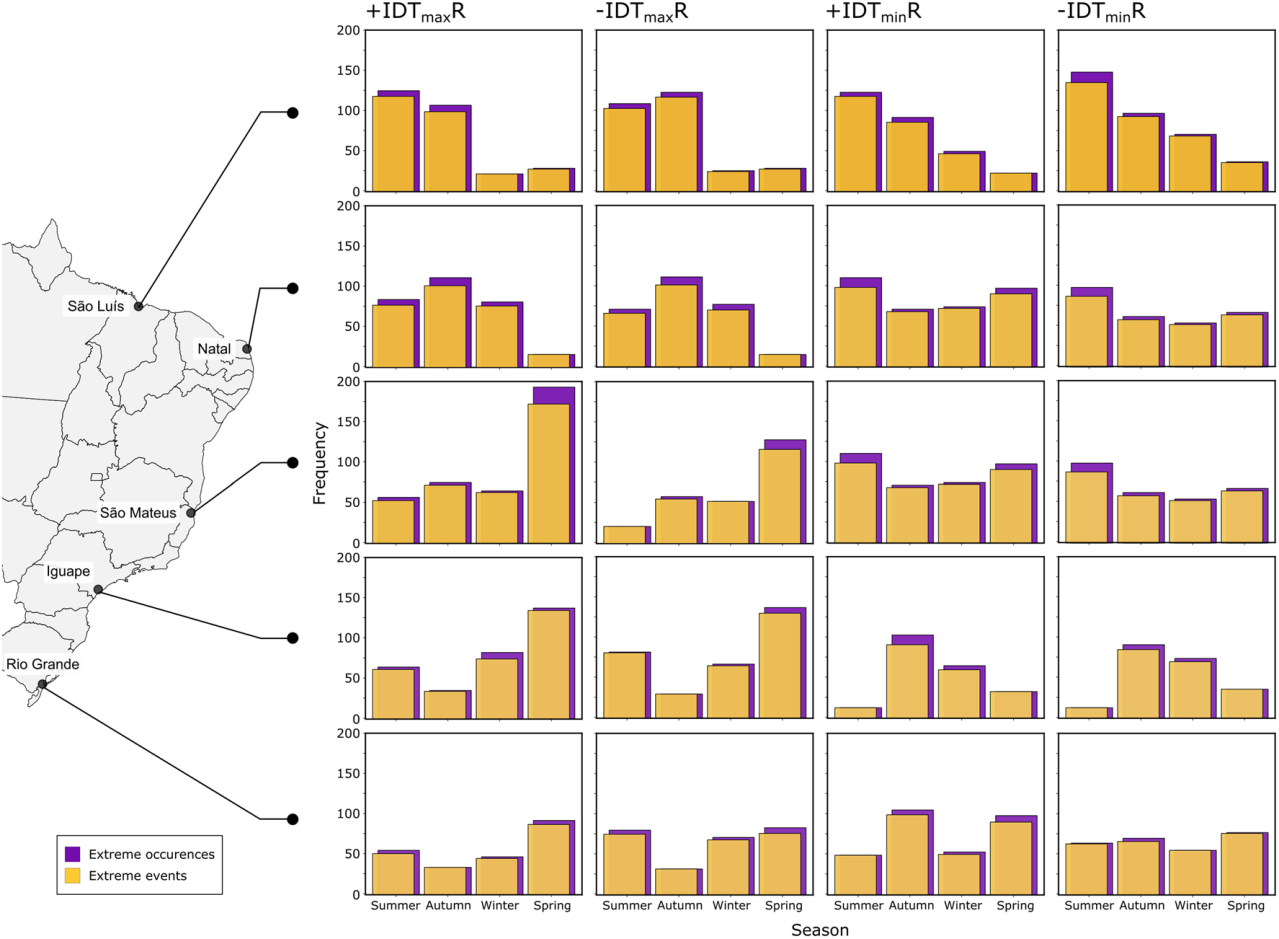
Overall seasonal frequency of extreme occurrence and events from all 5 MEOW in the last 40 years. The results are presented to the following response variables: positive inter-daily T_max_ range (+ IDT_max_R); positive inter-daily T_min_ range (+ IDT_min_R); negative inter-daily T_max_ range (− IDT_max_R); and negative inter-daily T_min_ range (− IDT_min_R).

Next, we will present the seasonal frequency of extreme occurrences and events along all 5 marine ecoregions on the Brazilian coast. The results are also presented herein as a comparison between those ecoregions for each response variable (Fig. 3: T_max_, T_min_, and DTR; Fig. 4: + IDT_max_R, + IDT_min_R, − IDT_max_R, − IDT_min_R). See Ref.^52^ for the detailed results per region.

Regarding the T_max_ and T_min_ extreme occurrences and events in São Luís, they were more frequent in Summer, similar to the T_min_ in Natal, while the T_max_ frequency was higher in Autumn (followed by Summer) for the last one. In contrast, Summer was the season with lower extreme occurrences and events for both T_max_ and T_min_ in São Mateus, while Spring and Winter was the one with the higher values, respectively. In Iguape, both extreme occurrences and events of T_max_ also occurred the most in Spring, similar to the Rio Grande extreme events, although extreme occurrences were more frequent in Summer (with a close frequency between Summer, Spring, and Winter). For the T_min_ extreme occurrences and events from both areas, they were more frequent in Autumn. In contrast, the DTR extreme occurrences and events in São Luis, Natal, and São Mateus, occurred the most in Winter, while in Iguape and Rio Grande in Spring (followed by Winter in Iguape, but Summer in the Rio Grande).

Concerning the climate amplitude in between consecutive days (cooling situation: + IDT_max_R and + IDT_min_R; warming situation: − IDT_max_R and − IDT_min_R), it was found that to the + IDT_max_R, + IDT_min_R, and − IDT_min_R in São Luís, Summer was the season with the higher frequency of extreme occurrences and events. This is similar to Natal for + IDT_min_R and − IDT_min_R, while + IDT_max_R extreme occurrences and events were more frequent in Autumn. This is also the season with greater occurrences and events from both São Luís and Natal for the − IDT_max_R. Differently, the southern sites of São Mateus, Iguape, and Rio Grande had a greater frequency of extreme occurrences and events in Spring for + IDT_max_R and − IDT_max_R (also − IDT_min_R for Rio Grande), while the + IDT_min_R and − IDT_min_R were more frequent in Winter in São Mateus, and in Autumn in Iguape.

### Monthly overview of extreme temperature

**Figure 5:**
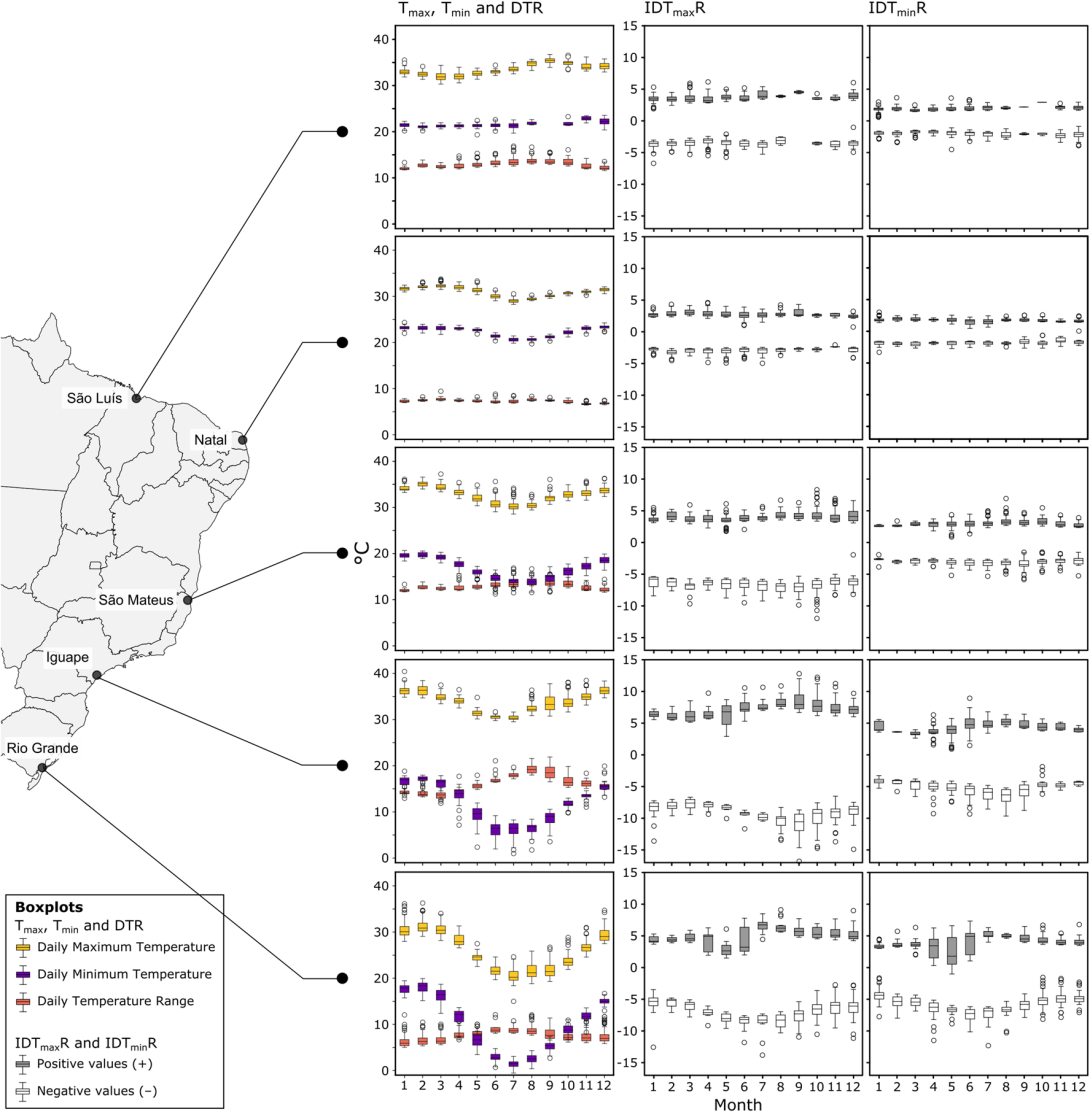
Overall monthly overview of temperatures considered extremes from all 5 MEOW in the last 40 years. The results are presented to the following response variables: daily maximum temperatures (T_max_); daily minimum temperatures (T_min_); daily temperature range (DTR); positive inter-daily T_max_ range (+ IDT_max_R); positive inter-daily T_min_ range (+ IDT_min_R); negative inter-daily T_max_ range (− IDT_max_R); and negative inter-daily T_min_ range (− IDT_min_R).

Finally, we will present herein the monthly overview of temperatures considered extremes for each region in the last 40 years. It is important to attest that extreme thresholds identified by the POT method provide extreme SAT values that change in time according to the monthly data distribution; thus, the results presented herein help get comprehensive insights on temperature extremes based on observational data throughout the evaluated timeframe. The results are also presented as a comparison between those ecoregions for each response variable (Fig. 5: T_max_, T_min_, DTR, + IDT_max_R, + IDT_min_R, − IDT_max_R, and − IDT_min_R). See Ref.^52^ for the detailed results per region.

In São Luís, the lower T_max_ extreme temperatures ranged from 30.3 °C, observed in March 1985, to 36.8 °C, observed in September 2011. The T_min_ changed from 19.3 °C in May 1988, to 23.5 °C in December 2010. Natal presented a similar temperature range, although the extremer T_max_ was neither too high (from 28.2 °C in July 1992 to 33.7 °C observed in March 1988) nor the extremer T_min_ too low (19.7 °C, in August 1986, to 24.3 °C, in January 1999). Furthermore, the seasonal pattern is opposite to both sites, since in São Luís the higher temperatures were observed in Winter/ Spring and lower in Summer/ Autumn (Natal had higher temperatures in Summer and lower in Winter). For the other 3 southern regions, the seasonal temperature extremes pattern is similar to Natal, although the overall variability of temperature extremes, as well as the variability in the same month (boxplot widths in Fig. 5), is higher in the southward sites. São Mateus had extreme T_max_ (ranging from 28.6 °C, in July 1987, to 37.2 °C, in March 2013) and T_min_ (from 11.2 °C in June 1993 to 20.7 °C in January 2016) than both northern regions. In Iguape, it was found the higher extreme T_max_ (varying from 29.5 °C, seen in July 2000, to 40.4 °C, observed in January 2016) and greater variation in T_min_ (the lowest was 1.0 °C in July 1990 and the highest was 17.9 °C, observed in February 2018), while in Rio Grande it was found the lowest extreme T_min_ (from − 0.5 °C in July 2007 to 19.9 °C, observed in February 2018), with T_max_ ranging from 18.4 °C, seen in July 1994, to 36.3 °C, observed in February 2010.

To the climate amplitude on the same day or in between consecutive days, we also found a latitudinal pattern. Natal was the region with lower amplitude to all response variables (DTR: 9.4 °C in March 1988; + IDT_max_R: 4.6 °C in April 1985; + IDT_min_R: 3.0 °C in January 1989; − IDT_max_R: − 4.9 °C in July 2018; and − IDT_min_R: − 3.3 °C in January 1999), followed by São Luís (DTR: 13.6 °C, in August 2012; + IDT_max_R: 6.1 °C in April 1996; + IDT_min_R: 3.6 °C in May 1988; − IDT_max_R: − 6.7 °C in January 1999; and − IDT_min_R: − 4.5 °C in May 2010). In both regions, there is not a clear seasonal pattern of extreme temperatures. Also, the overall variability of temperature extremes, as well as the variability in the same month (interquartile range in Fig. 5) is lower than in the 3 southern sites. In these areas, the temperature amplitude is higher (both positive and negative values) in Winter/ Spring. Iguape is the region with extremer values to almost all response variables (DTR: 17.9 °C, observed in February 2018; + IDT_max_R: 12.8 °C, from September 1997; + IDT_min_R: 8.9 °C in June 2004; − IDT_max_R: − 16.8 °C in September 2006; and − IDT_min_R: 9.6 °C in August 1991), followed by São Mateus (DTR: 16.9 °C in July 2019; + IDT_max_R: 8.4 °C, from October 2016; + IDT_min_R: 6.9 °C in August 1993; − IDT_max_R: 11.9 °C in October 2011; and − IDT_min_R: − 5.8 °C in September 2001), and Rio Grande, where the only variable with extremer values is the − IDT_min_R (DTR: 15.0 °C from July 2017; + IDT_max_R: 9.1 °C, from August 2006; + IDT_min_R: 7.3 °C in June 1982; − IDT_max_R: − 13.8 °C in July 2017; and − IDT_min_R: − 12.3 °C in July 2017).

## Discussion

The IPCC has been highlighting climate extremes since its report in 2012^26^ and also, more recently, in the sixth assessment report^1^. Overall, they attest that human-induced climate changes are increasing the frequency and intensity of extreme temperatures (e.g., cold temperatures are less frequent and intense). It is expected that the frequency of these extremes will increase with each extra 1 °C of global warming. All modeled predictions are pointing to 1.5 °C of global warming by 2050, even the scenario with a negative carbon emission balance by 2055 (lower emission scenario), the only one that temperatures will decrease by 2100^1^. In coastal regions, one of the main concerns is the sea level rise, which has been rising faster in the last 100 years than in any other century over the last 3000 years^54^. Projections of extreme sea levels that occur once per century point to an increase of around 160 to 530 times by 2100^54^. Additionally, although the ocean is vital in regulating the climate by absorbing and storing the heat excess, reducing the rate of SAT warming^25^, our findings corroborate the concern about climate extremes in coastal areas. We have demonstrated an increase in the intensity and frequency of SAT extremes along the Brazilian coast, with amplified effects in higher latitudes. Given the potential effects of extreme climates on society and natural systems over the world^1,19,26,55^, our study points out the urge for action to mitigate the effects of the increase in SAT extremes in the coastal zones.

Methodologically, data quality is an increasing challenge in climate research^56^. Some areas of the globe are well covered, such as Europe^57,58^ and North America^11^, but in some regions, data scarcity can be an issue. For these regions, a reanalysis dataset, a hybrid model observation produced by assimilating ground and satellite observations with climate model simulations, can be an alternative solution^55^. Biases in the reanalysis models can be particularly important for coastal regions since the nearest grid point can be in the sea or a few kilometers inland from the coast, greatly influencing the SAT temperature estimates. In this sense, a site-adaptation technique has an important role in comparing and combining the long-range reanalysis data series with a not-too-long period of ground observational data to get reliable climatological time series representing the local climate pattern and variability.

Besides the database, the method to evaluate extremes is also critical. The POT method from the EVT has been demonstrated to be a useful tool for evaluating extremes in many areas, such as hydrology^59^, material corrosion^60^, finance, insurance, or other fields^14^. In the climate change context, it has been extensively used to evaluate extremes in precipitation^58^, wind speed^61^, wave height^61,62^, and also SAT temperatures^58,63^. The main purpose of the EVT is to describe the rare events in data distribution by the distribution of the generalized extreme value (GEV) and/or the generalized Pareto distribution (GPD)^15,16^. The first one fits the series from block maxima^59^, while the GPD fits the data provided by the POT^59^. Although the POT method contains a subjective threshold selection, it is frequently used due to its efficiency in describing the extremes^58^. In this sense, our study reinforces that this can be an excellent method to make inferences related to the intensity of extremes, by observing the exceedance over the threshold series, the frequency of extreme episodes, by observing the time elapsed between them, and the number of extremes per year. Applied to the series of deviations from the normalized 40-year monthly mean, it can also be advantageous for predicting seasonal variations of climatic extremes, allowing us to know at what time of year these extremes tend to be more common and close to values that can cause a great impact, such as flooding related to heavy rains, extreme heat, or coastal storms. Given the explanation of extreme conditions based on occurrences that exceed a certain threshold, our definition of “extreme occurrence” is a good approach to evaluating extreme intensity. In contrast, “extreme event” is related to the duration of extremes, which can be useful in the heat waves context, for example with three or more consecutive days with extreme occurrences^64,65^.

Undeniably, there are several studies describing “extremes” differently. For example, The Expert Team on Climate Change Detection and Indices (ETCCDI) has defined some extreme indices which have been applied worldwide, as well as in Brazil^36,66,67^. Some indices are based on fixed thresholds, which can be the same for different places. Alternatively, some indices are based on thresholds defined as a percentile from a data series, which vary depending on the location. The idea to standardize those indices is that they can be a common basis for the assessments of climate change in different studies and reports^66,68,69^. We believe that they are extremely important and have positively added to the understanding of climate change. Nevertheless, the extreme indices based on fixed thresholds might not make sense to be used in some parts of the globe (e.g., the number of temperatures below 0 °C or above 25 °C in a tropical region). In addition, the indices based on thresholds defined as a percentile from a data series are not evaluated based on a specific distribution for extreme values, but on the tail of a series of an observed historical dataset. Herein, we identified extremes in the time series of deviation values to the monthly average, by adjusting a probabilistic distribution for the extreme values (and not taking the highest values of a historical series verifying how many times occurred per unit of time). The POT establishes a threshold not for temperature values, but for values of temperature deviations from the 40-year average for that month. A final regard is that it is also possible to estimate the return period and the return level, although we did not explore this tool in this paper. Therefore, we believe that, given all the explanations above of the advantages of the method used in this study, the Environmental Sciences can benefit a lot from the EVT approach, which has been widely used in areas such as finance and insurance in risk analysis. We also believe that both methods have their advantages and disadvantages and, thus, they can be complementary.

Herein, our results indicate that extremes of SAT are becoming more intense and more frequent over the last 40 years throughout all 5 MEOW from the Brazilian coast, but the duration of extreme events is barely affected. Overall, the Brazilian coast has a large latitudinal extension with great climatic variability^29^. The northern coast is mainly influenced by the warm Equatorial North Atlantic air mass (Ean) during summer, while in rainy months by the colder/showery weather Equatorial South Atlantic air mass (Eas) ^31,32,70^. São Luís climate extremes occurrences and events of T_max_ and T_min_ have not increased in intensity, frequency, or duration throughout time, similarly to what was observed in Natal. Interestingly, in São Luís, both T_max_ and T_min_ extremes occurred the most in summer, probably associated with the Ean. The same pattern was found for T_min_ in Natal, while T_max_ occurred the most in Autumn (followed by summer), possibly related to the transition from the Ean to Eas in the rainy season in this area^71^. In contrast, we found a different pattern concerning climate variability on the same day (DTR) or between consecutive days (IDTR). Both had an increasing trend of frequency in São Luís, regardless of whether it is a warming or cooling meteorological condition (i.e., both positive and negative values of IDTR). Also, São Luís was the only location that presented an increase in the event duration of extreme DTR and the + IDT_max_R (i.e., cooling situation). In Natal, we only observed an increasing trend in the frequency of extreme events and occurrences for the IDTR (in both cooling and warming situations), suggesting that this region has a climate less susceptible to the increase in the frequency of climate extremes than the previous one. Extremes in the DTR were more common in winter in both regions, probably related to the thermodynamically cool Eas, while summer was the season that showed an increase in the frequency of extremes of all indicators, even for the IDTR that attests to both warming and cooling conditions. This suggests that the summer warmer days related to Ean, but not the cold ones, can play a role in extreme temperature change within days for both locations.

Alternatively, the southern coast is influenced by the warm Tropical Atlantic air masses (Ta) and by the cool Polar Atlantic air mass (Pa)^31,32,70^. São Mateus demonstrated an increasing trend in the frequency of extreme occurrences and events of both T_max_ and T_min_; Iguape showed an increasing trend in the frequency of extreme occurrences and events to T_max_, but not to T_min_; while Rio Grande showed not only an increasing trend on the frequency to both occurrences and events of T_max_, but it is the only location with an increasing trend in the intensity of T_min_. For all three sites, the T_max_ extremes occurred the most in spring, probably associated with the warm Ta, while extremes in T_min_ were more common in winter/autumn, a time of the year in which cold fronts, an atmospheric system related to Pa, are usual^20,72^. São Mateus, Iguape, and Rio Grande also demonstrated not only an increasing trend in the extreme frequency of the DTR index but on its intensity. Nevertheless, there was no effect on any extreme indicators related to the IDTR in São Mateus, while Iguape and Rio Grande showed an increasing trend in the frequency of extreme occurrences and events only for one cooling situation (+ IDT_max_R). The DTR extremes were also more frequent in winter months in all three sites, probably associated with the influence of cool Pa in this season at this region, while IDTR extremes occurred the most in spring, a season that the number of cold fronts is reduced compared to the winter^20,72^. Generally, there is a phenomenon called prefrontal heat around a day or a few hours before a cold front. The hot air is stalled by the cold air mass that arrives with the front, causing even greater heating of the air in this region. When the cold front arrives, the cold air mass displaces the warm air, causing wind shifts, a drop in atmospheric pressure, and rain formation^20^. Due to its seasonal pattern, the use of the DTR index seems to be a better approach to make inferences about air mass changes than the IDTR, but joint analyses on extremes with other atmospheric variables are desirable, such as air pressure, precipitation, or wind changes. In addition, the southern sites were the ones with higher extremes of T_max_ (Iguape), lower extremes of T_min_ (Rio Grande), extremer DTR, and almost all variables of the IDTR, and also higher overall variability of SAT extremes (see boxplots widths in Fig. 5). Therefore, there is a clear gradient of extremes throughout the latitude, following the settled perception that areas in higher latitudes will be more affected by the extent of warming^73^, but in contrast to the global emergence of extreme heat increase in the low-latitude^6^.

Many studies using different approaches have shown an increase in some climate extremes indexes for different response variables worldwide. For example, in some countries in South and West Africa, warm days and nights have become more frequent, while it was the opposite for cold days and nights^74,75^. Also, there are consistent increases in DTR, coinciding with increases in maximum temperature extremes^74^. In China, it was found that cold extremes are warming faster than hot extremes^76^. Over non-urban places in the Iberian Peninsula, there was an increasing trend of extreme temperatures for both T_min_ and T_max_^63^, consistent with earlier studies with the threshold considered by a different method^77^. Approaches other than the commonly used trend analysis showed an increase in synchronized droughts and heatwaves over some parts of the United States, with shifts in the distribution of concurrent extremes^11^. There was also an increase in heat wave occurrences in eastern Australia and South Australia since 1950^64^, along with impacts of extreme climate events (which are unique in intensity and are becoming more frequent) affecting corals, seagrasses, kelps, and mangroves around Australian coast^6^. In the entire tropical South America, some future projections are attesting that extremely warm nights will be more frequent, while cold night events are likely to decrease^4^. Some projections suggest an increase in precipitation during the rainy days in some parts of Brazil, including the coastal region^78^, and many other studies with different methods and extreme indexes attest to increased extremes in different parts of Brazil^71,79,80^. In general, most of the studies on extreme events point to the global effects of these climate extremes nowadays and the increased challenge we will face in the near future. Although these negative scenarios, existing approaches to dealing with climate extremes should be constantly reviewed, taking into consideration the continuous updating of scientific tools, and the practical adaptation and planning strategies^81^.

In summary, we found that extreme occurrences of SAT are becoming more intense and more frequent over the last 40 years throughout all 5 MEOW from the Brazilian coast, with increased effects in higher latitudes. Also, the DTR patterns can be essential to help identify extreme cold fronts, although a joint analysis with other response variables, such as air pressure and precipitation, is desirable. Despite having different techniques for identifying climate extremes, the results found herein are following the studies worldwide. Although using the same methods of evaluating extremes can be important to facilitate the comparison between areas^82^, we argue that it is also essential to use different methods in a complementary manner, along with the continuous update of scientific tools. Finally, the dataset and the method used herein seem to be a reliable approach for the studies of climate extremes, with clear indicators of its intensity, frequency, and duration, as well as seasonal details. It can be easily applied to other regions of our planet in a wide range of topics, which can be worthwhile for extreme risk management.

## Data Availability

All supplementary files are available in the figshare repository. Observed dataset: 10.6084/m9.figshare.21648773; reanalysis dataset: 10.6084/m9.figshare.21648749. Detailed results from bias-removing analyses: 10.6084/m9.figshare.21648788; scripts codes from bias removing analyses: 10.6084/m9.figshare.21650951. Detailed results from all extreme analyses: 10.6084/m9.figshare.21649322; scripts codes from all extreme analyses: 10.6084/m9.figshare.21650909.
